# Editorial: Recombinant microbes-mediated cancer immunotherapy

**DOI:** 10.3389/fimmu.2024.1386574

**Published:** 2024-02-22

**Authors:** Stephen Wechman, Venkatakrishna Rao Jala, Luis G. Bermudez-Humaran, Jorge G. Gomez-Gutierrez

**Affiliations:** ^1^ Union College, Barbourville, KY, United States; ^2^ Department of Microbiology and Immunology, University of Louisville, Louisville, KY, United States; ^3^ National Research Institute for Agriculture, Food and Environment, Jouy-en-Josas, France; ^4^ Department of Pediatrics, University of Missouri-Columbia, Columbia, MO, United States

**Keywords:** recombinant, microbes, cancer, immunotherapy, treatment

Cancer remains one of the major challenges of the 21st century. The increasing numbers of cases are not accompanied by adequate progress in therapy. The standard methods of treatment often do not lead to the expected effects. Therefore, it is extremely important to find new, more effective treatments. One of the most promising research directions is immunotherapy, including the use of specific types of microorganisms. This type of treatment is expected to stimulate the immune system for the selective elimination of cancer cells. It is possible to use microorganisms in many ways, based on their specific properties, that is, toxin production, anaerobic lifestyle, or binding substances that can be delivered to a specific location (vectors).

The main priorities in cancer research are prevention, early detection, and the development of new therapies, including personalized therapies, which are intended to include the molecular biology of a particular tumor and the predisposition of the patient’s immune system. Among the known and practiced anticancer therapies, the use of microbes appears to be one of the most promising strategies. Although now somewhat forgotten, it has a large potential to play a significant role in the treatment of cancer. Thus, microbial-based cancer immunotherapy aims to use tumor-specific infectious microbes to fulfill the unmet medical needs for patients with difficult-to-treat malignancies.

This Research Topic focuses on the interaction of microbe-tumor and discusses recent advances in the field that take advantage of the unique ability of microbes to invade human cells and induce immune responses to create therapeutic approaches that direct microbes to selectively target tumors.

In this regard, S100A8 a protein with reported pro- and anti-inflammatory functions was repeatedly administered intranasally to mice bearing orthotopic lung cancers. S100A8 treatment prolonged survival from 19 days to 28 days. It was found that S100A8 significantly lowered expression of cytokine genes and proteins that promote expansion and activation of myeloid-derived suppressor cells (MDSC). Furthermore, S100A8 reprogramed the lung microenvironment allowing cytotoxic lymphocyte expansion and function, decreased MDSC numbers and increased numbers of CD4+ T cells and natural killer T (NK-T) cells. These results indicate that S100A8 modify the lung microenvironment to promote an effective immune response in the lungs (Wong et al.).

Another study found that animals immunized with either vaccine, HPV16 RG1-VLP or CUT-PANHPVAX developed cross-reactive antibodies against the cutaneous Mastomys natalensis papillomavirus (MnPV) L2, which were cross-neutralizing MnPV pseudovirions in vitro. Furthermore, this vaccination strategy prevented skin tumor formation and even microscopical signs of MnPV infection in the skin. This study provides fundamental insights into the humoral immunity elicited by L2-based vaccines against PV-induced skin tumors, with important implications to the design of next generation HPV vaccines (Ahmels et al.).

Recently the combined treatment with αPD-L1 mAb and Salmonella resulted in 75% inhibition of tumor growth in 100% of animals. Mechanistically, the enhanced response correlated with a decrease in the percentage of tumor-associated granulocytic cells, upregulation in MHC class II expression by intratumoral macrophages and an increase in tumor infiltration by effector T cells. This study demonstrates that a novel combination treatment utilizing attenuated Salmonella and αPD-L1 mAb could improve the outcome of immunotherapy in poorly immunogenic tumors (Al-Saafeen et al.).

A Listeria (Lm)-based vaccine encoding an antigenic fragment of CD105(Lm-LLO-CD105A) reduced primary tumor growth in both subcutaneous and orthotopic models of murine renal cell carcinoma (RCC). The vaccine conferred anti-tumor immunity and remodeled the tumor microenvironment (TME), resulting in increased infiltration of polyfunctional CD8+ and CD4+ T cells and reduced infiltration of immunosuppressive cell types within the TME. The finding in this study suggest that Lm-based immunotherapy is safety and promising therapeutic strategy for RCC (Oladejo et al.).

In another study it was found that treatment of 4T1.2-HER2T tumors with VSVΔ51+T-DM1 virus yielded robust curative efficacy compared to controls, and broad immunologic memory against the tumors. Interrogation of anti-tumor immunity revealed tumor infiltration by CD4+ T cells, and activation of B, NK, and dendritic cell responses, as well as tumor-reactive serum IgG. These data also suggest that the HER2T platform may be used to assess a range of surface-HER2T targeting approaches, such as CAR-T, T-cell engagers, antibodies, or even retargeted oncolytic viruses (Taha et al.).

An interesting strategy consisting of two-plasmid, tetracycline-inducible CRISPR-Cas9 system was used to create recombinant strains of Clostridium sporogenes expressing pro-inflammatory cytokines (IL-2 and GM-CSF) and a pro-drug converting enzyme. A comparative, temporal *in vitro* analysis of these strains revealed a substantial reduction of cytokine activity in chromosome-based constructs. To compensate for this loss, a 7.6 kb operon of proteolysis genes was deleted from the interegenome. Knockout strains showed an 8-to 10-fold increase in cytokine activity compared to parental strains (Kubiak et al.).

This Research Topic concludes with a remarkable finding showing that pre-treatment with dimethyl fumarate (DMF) or other fumaric and maleic acid esters (FMAEs) leads to a significant increase in viral growth of oHSV-1 in several cancer cell lines, including melanoma, while decreasing cell viability. Additionally, DMF was able to enhance ex vivo oHSV-1 infection of mouse-derived tumor cores as well as human patient tumor samples but not normal tissue. The increased viral spread and oncolysis of the combination therapy occurs via inhibition of type I IFN production and response. This study demonstrates the synergistic potential of two approved therapies for clinical evaluation in cancer patients (Alwithenani et al.).

## Author contributions

JG-G: Writing – original draft. SW: Writing – review & editing. VJ: Writing – review & editing. LB: Writing – review & editing.

